# Food allergy training event for restaurant staff; a pilot evaluation

**DOI:** 10.1186/2045-7022-4-26

**Published:** 2014-08-28

**Authors:** Samuel Bailey, Tiffany Billmeier Kindratt, Helen Smith, David Reading

**Affiliations:** 1 Division of Primary Care and Public Health, Brighton & Sussex Medical School, Brighton, UK; 2 Division of Medical Education, Brighton & Sussex Medical School, Brighton, UK; 3 Head of Division of Primary Care and Public Health, Brighton & Sussex Medical School, Brighton BN1 9PH, UK; 4 Anaphylaxis Campaign, Farnborough, Hampshire, UK

**Keywords:** Food allergy, Restaurant staff, Knowledge

## Abstract

A previous cross-sectional survey highlighted that restaurant staff in Brighton had gaps in their knowledge of food allergy, which could lead to the provision of unsafe meals to food-allergic customers. A food allergy training event was developed by a multi-disciplinary team (health service researcher, clinician, teacher and patient group representative) to equip restaurant staff with the knowledge and skills necessary to safely serve food-allergic customers. This evaluation summarises the training event’s impact on participants’ knowledge of food allergy and their satisfaction with the event.

No attendee had previously attended any formal training on food allergy. The percentage of participants who answered all true-false questions correctly increased from 82% before the training event to 91% afterwards. The percentage of participants who were able to name at least three common allergens increased from 9% to 64%. Both quantitative and qualitative feedback was positive.

Restaurant staff require a good understanding of food allergy to ensure that food-allergic customers are kept safe, and their restaurants operate within the law. This food allergy training event improved participants’ absolute knowledge of food allergy, and attendees changed practice. Recommendations are made which could improve the impact and uptake of future food allergy training events.

## Findings

### Introduction

Previous studies have highlighted worrying gaps in restaurant staffs’ knowledge of food allergy, which challenges how capable they are of delivering a safe meal to a food allergic customer [1-3]. All restaurant staff are involved in the process, whether it be the waiter describing food accurately and ascertaining the customer’s needs, the supervisor communicating effectively with the kitchen to confirm their awareness of the request, or the chef ensuring that the allergen is avoided. A weakness in any staff member in food allergy can result in unsafe food being served.

In the UK all restaurant staff are required to be trained in food hygiene, with the level of participation depending upon the employee’s role within the restaurant. The higher-level formal food hygiene courses, aimed at staff who prepare ready-to-eat foods or have a supervisory role, include some elements of food allergy training [4]. However, the standard training for servers does not include food allergy. Training courses specifically about food allergy are available, but participation is voluntary. We designed a brief educational intervention and evaluated its impact on participants’ knowledge of food allergy and the participants’ satisfaction.

## Methods

### Recruitment of training participants

Contact details for all dine-in restaurants in Brighton, UK were identified from two online company databases (http://www.thomsonlocal.com and http://www.yell.com). The manager of each restaurant was sent an invitation to nominate one or more employees for free allergy training. The invite referenced an earlier study in the city which identified gaps in restaurant staffs’ knowledge of food allergy [1].

### Food allergy training

The training was developed as a one-hour lecture. The learning outcomes were informed by a local survey highlighting deficits in restaurants staff’s knowledge of anaphylaxis and dietary care of people with allergies [1]. It was intended that by the end of the training participants should be able to:

List the most common UK food allergens.

Understand the differences between food allergies and food sensitivities and intolerances.

Be aware how to avoid allergen exposure (including use of food labelling policies and avoidance of contamination).

Recognise the symptoms of severe food allergic reactions and anaphylaxis.

Respond safely and appropriately if an allergic reaction occurs in one of their customers.

Communicate effectively with food-allergic customers and their guardians to ascertain their dietary needs.

The resources were created by an officer from the UK-charity Anaphylaxis Campaign (DR), an academic clinician with a special interest in allergy (HS) and a health service researcher (TBK). The intervention was piloted with 10 chefs working in University kitchens. The training event was provided at a venue in the city centre and was delivered by DR. Participants were given a certificate of attendance on completion of the course.

### Data collection

Data were collected immediately before and on conclusion of the training to assess change in participant’s knowledge of food allergy. Participants completed a questionnaire which asked them to list three common food allergens and respond to six true-false questions. The Wilcoxon signed-rank test was used to determine significant changes in knowledge scores.

Participants understanding of allergy, the relevance of training to practice, their confidence dealing with a food allergy emergency and their satisfaction with the training was evaluated using a 5-point Likert scale (1 = strongly disagree to 5 = strongly agree). Participants were also asked to comment on what they most liked about the course, how it could be improved and what they will do differently at work as a result of their training.

Four weeks later, a further test questionnaire of knowledge was distributed and further qualitative comments were invited. Responses could be sent by mail or via an online survey.

Ethical approval for this research project was granted by Brighton and Sussex Medical School Research Governance and Ethical Committee (12/039/SMI).

## Results

### Response and respondents’ demographics

The database search identified 189 dine-in restaurants. Fifteen participants from six restaurants registered for the training and 11 participants from four restaurants attended. Participants were predominantly male (64%), mean age 33 years (range 18 to 58) who worked full time (91%) and had worked in the catering industry for an average of 7.8 years (range 0.5 to 20 years). Six staff were front of house (manager or waiters) and 5 worked in the kitchen (chef, kitchen assistant), none had undergone previous food allergy training. Participants came from Asian, European, Mexican and vegetarian restaurants all situated close (0.3 to 1.0 km) to the venue.

### Knowledge about food allergy

Before the training, nine participants (82%) correctly identified that all six statements were false (Table 1). Incorrect answers were documented by two participants (18%), who both believed that a customer having an allergic reaction should be served water to ‘dilute’ the allergen. One of these participants (9%) also thought that a clean buffet counter containing allergens can be safely used by a food allergic customer. After the training, participants’ responses to these statements improved. Ten participants (91%) answered all true-false questions correct but one participant continued to believe that a customer having an allergic reaction should be served water to ‘dilute’ the allergen.

**Table 1:** Restaurant staff’s knowledge of food allergy (n = 11)

	**Disagreeing with statement, count (%)**		
	**Pre-training**^**1**^	**Post-training**^**1**^	**4-week follow-up quiz**
**Statement**	**n = 11**	**n = 11**	**n = 3**
Individuals with food allergies can safely consume a small amount of that food (false)	11 (100)	11 (100)	3 (100)
High heat (e.g. oil frying) can destroy most food allergens (false)	11 (100)	11 (100)	3 (100)
If a customer is having an allergic reaction, it is appropriate to serve them water to “dilute” the allergen and suppress the reaction (false)	9 (82)	10 (90)	3 (100)
If a buffet (serve-yourself) counter contains allergens but is kept clean, it can be a safe choice for a food allergic customer (false)	10 (91)	11 (100)	3 (100)
Removing an allergen from a finished meal (e.g. taking off nuts) may be required to provide a safe meal for a food allergic customer (false)	11 (100)	11 (100)	3 (100)
Food allergy and food intolerance means the same thing (false)	11 (100)	11 (100)	3 (100)

^1^The overall improvement in participants’ knowledge of food allergy before and after the training event was not statistically significant (p = 0.074).

Before the training only one participant (9%) was able to list 3 of the 8 common food allergens (tree nut, peanut, egg, milk, fish, shellfish, wheat, soy). Awareness of common food allergens improved after the training, with seven participants (64%) then able to name three common allergens. The three participants (27%) who responded to the follow-up quiz at 4-weeks answered all true-false questions correctly, and successfully named three common food allergens.

### Satisfaction with training

Participants rated the training program favourably (Table 2). Participants agreed most with the statement, ‘I will use what I learned in the course to change my food handling practices to prevent a food allergic reaction’ (mean = 4.18) and this applicability to their restaurants was reflected in their free text comments. Participants valued the clarity and accessibility of the training but wanted more depth of knowledge and a longer time.

**Table 2:** Participant’s quantitative and qualitative feedback immediately after training (n = 11)

**Scores on Likert scale (1 = strongly disagree to 5 = strongly agree)**	
**Statement**	**Mean (SD)**
I have a better understanding of food allergens after taking this course	3.91 (0.83)
I will use what I learned in this course by changing my food handling practices to prevent a food allergic reaction	4.18 (0.87)
I am confident that I can handle a food allergic emergency	4.09 (0.94)
I understand safe food handling practices to prevent cross-contact of food allergens	4.09 (0.83)
Overall, I was satisfied with the quality of the course	4.09 (0.83)
**Verbatim responses to open ended questions**	
Question	
**What did you like best about the training?**	
‘Clear information’, ‘Easy to understand’, ‘Short and clear’, ‘Informal and accommodating lecturer’	
**How can the training be improved?**	
‘More knowledgeable information’, ‘Too basic’, ‘Longer training’, ‘Rushed at the end’	
**When you go to work next time, what will you do differently since coming to this training?**	
‘Wash hands well’, ‘Be more aware of allergies for customers’, ‘Check all ingredients of products used in my restaurant’, ‘Make more prominent signs, train staff and update recipe book’, ‘Make/serve food to customers with allergies separately from other customers on the table’	

At four weeks feedback was very encouraging with adjustment reported in the kitchen (e.g., ‘we have carefully listed all ingredients in a recipe book that all the staff can refer to’ , ‘separated some cooking tools for allergic customers to prevent cross-contamination’) and in the front of house (‘now successfully liaise with customers with allergies in order to select dishes suitable for them to safely eat’ , ‘put extra notices up asking customers to let us know of any allergy or intolerance’ ‘[we] write down clearly in the menu about the chance of allergy to food’.

## Discussion

To our knowledge, this is the first evaluation of a food allergy training event in the United Kingdom. Although a small event, we feel it important to share our results and outline our recommendations to guide future training. The training event improved participants’ knowledge, changed practice, and attendees were generally satisfied.

The number of attendees was low. In our previous study, which prompted the provision of this training event, 48% of 90 restaurant staff surveyed in Brighton expressed an interest in further food allergy training [1]. There may be a number of factors that contributed to poor uptake. Firstly, the letters of invitation were sent to the restaurant managers rather than to individual members of staff. Whilst we piloted our teaching materials we did not pilot either the recruitment materials or questionnaires. The training was provided free of charge, however it would have opportunity costs for the restaurant and their staff’s time.

The training did not appear to have reached those most in need as the pre-training scores were relatively high. With voluntary participation attendees are more likely to be enthusiastic staff, perhaps motivated by first-hand experience, from restaurants where food allergy is taken seriously rather than those where practice had been identified to be poor. By basing our training around the educational needs identified in a city wide survey [1] we underestimated the food allergy knowledge of some participants. In future we will adopt a less didactic training style, identifying participants’ learning needs at the outset so that the presentation can be tailored to their specific needs. Some participants commented their preference for a longer training session, this would enable us to introduce some active learning elements to the training, for example small group work around communication with food allergic customers, a feature which may have reinforced learning and improved participants’ satisfaction [5].

The knowledge base of restaurant employees about allergy needs to be improved in a way that is relevant, cheap and accessible. We are considering organising events within restaurants as a restaurant provides a better environment to demonstrate practical issues such as avoiding cross contamination and clarity of menus. On-line food allergy training is another option and is well suited to reaching a disparate group of people often working outside standard hours. Another approach would be to train and accredit some restaurant staff as food allergy ambassadors and provide them with the resources to enable them to provide food allergy training events in their restaurant, for neighbouring restaurants in their locality. Whatever the training methods chosen, future initiatives need to optimise participation rates and assess whether the impact of training is sustained over a longer period.

The evaluation of future food allergy training events is recommended to enable us to develop effective and efficient ways of reducing the risks of dining out for the food allergic customer.

## Competing interests

The authors declare that they have no competing interests.

## Authors’ contributions

SB participated in the analysis and interpretation of the data and helped draft the manuscript. TBK contributed to the design of the educational intervention and co-ordinated the study, performing the statistical analysis and critical revision of the manuscript. HS conceived the study and contributed to the design of the educational intervention and drafting of the manuscript. DR designed and delivered the educational intervention. All authors read and approved the final manuscript.
